# Genetic Characterization of Human T-Cell Lymphotropic Virus Type 1 in Mozambique: Transcontinental Lineages Drive the HTLV-1 Endemic

**DOI:** 10.1371/journal.pntd.0001038

**Published:** 2011-04-12

**Authors:** Ana Carolina P. Vicente, Eduardo Samo Gudo, Alena Mayo Iñiguez, Koko Otsuki, Nilesh Bhatt, Celina M. Abreu, Adolfo Vubil, Dulce Bila, Orlando C. Ferreira, Amílcar Tanuri, Ilesh V. Jani, for the HTLV in Mozambique Study Group

**Affiliations:** 1 Instituto Oswaldo Cruz, Fundação Oswaldo Cruz (FIOCRUZ), Rio de Janeiro, Brazil; 2 Departamento de Imunologia, Instituto Nacional de Saúde, Maputo, Mozambique; 3 Departamento de Genética, Universidade Federal do Rio de Janeiro, Rio de Janeiro, Brazil; George Mason University, United States of America

## Abstract

**Background:**

Human T-Cell Lymphotropic Virus Type 1 (HTLV-1) is the etiological agent of adult T-cell leukemia (ATL) and HTLV-1-associated myelopathy/tropical spastic paraparesis (HAM/TSP). It has been estimated that 10–20 million people are infected worldwide, but no successful treatment is available. Recently, the epidemiology of this virus was addressed in blood donors from Maputo, showing rates from 0.9 to 1.2%. However, the origin and impact of HTLV endemic in this population is unknown.

**Objective:**

To assess the HTLV-1 molecular epidemiology in Mozambique and to investigate their relationship with HTLV-1 lineages circulating worldwide.

**Methods:**

Blood donors and HIV patients were screened for HTLV antibodies by using enzyme immunoassay, followed by Western Blot. PCR and sequencing of HTLV-1 LTR region were applied and genetic HTLV-1 subtypes were assigned by the neighbor-joining method. The mean genetic distance of Mozambican HTLV-1 lineages among the genetic clusters were determined. Human mitochondrial (mt) DNA analysis was performed and individuals classified in mtDNA haplogroups.

**Results:**

LTR HTLV-1 analysis demonstrated that all isolates belong to the Transcontinental subgroup of the Cosmopolitan subtype. Mozambican HTLV-1 sequences had a high inter-strain genetic distance, reflecting in three major clusters. One cluster is associated with the South Africa sequences, one is related with Middle East and India strains and the third is a specific Mozambican cluster. Interestingly, 83.3% of HIV/HTLV-1 co-infection was observed in the Mozambican cluster. The human mtDNA haplotypes revealed that all belong to the African macrohaplogroup L with frequencies representatives of the country.

**Conclusions:**

The Mozambican HTLV-1 genetic diversity detected in this study reveals that although the strains belong to the most prevalent and worldwide distributed Transcontinental subgroup of the Cosmopolitan subtype, there is a high HTLV diversity that could be correlated with at least 3 different HTLV-1 introductions in the country. The significant rate of HTLV-1a/HIV-1C co-infection, particularly in the Mozambican cluster, has important implications for the controls programs of both viruses.

## Author Summary

Human T-cell lymphotropic virus type 1 (HTLV-1) is the causative agent of Adult T-Cell Leukemia/Lymphoma (ATL), the Tropical Spastic Paraparesis/HTLV-1-associated Myelopathy (TSP/HAM) and other inflammatory diseases, including dermatitis, uveitis, and myositis. It is estimated that 2–8% of the infected persons will develop a HTLV-1-associated disease during their lifetimes, frequently TSP/HAM. Thus far, there is not a specific treatment to this progressive and chronic disease. HTLV-1 has means of three transmission: (i) from mother to child during prolonged breastfeeding, (ii) between sexual partners and (iii) through blood transfusion. HTLV-1 has been characterized in 7 subtypes and the geographical distribution and the clinical impact of this infection is not well known, mainly in African population. HTLV-1 is endemic in sub-Saharan Africa. Mozambique is a country of southeastern Africa where TSP/HAM cases were reported. Recently, our group estimated the HTLV prevalence among Mozambican blood donors as 0.9%. In this work we performed a genetic analysis of HTLV-1 in blood donors and HIV/HTLV co-infected patients from Maputo, Mozambique. Our results showed the presence of three HTLV-1 clusters within the Cosmopolitan/Transcontinental subtype/subgroup. The differential rates of HIV-1/HTLV-1 co-infection in the three HTLV-1 clusters demonstrated the dynamic of the two viruses and the need for implementation of control measures focusing on both retroviruses.

## Introduction

Human T-cell lymphotropic virus type 1 was the first oncogenic human retrovirus to be identified in 1980 [1] In 1982, the second type, HTLV-2, was discovered [2]. These two human viruses originated independently through zoonotic infections from lineages of simian T-lymphotropic virus (STLV-1 and STLV-2). These inter-species transmission events have been occurring in Africa up to recent times [3]–[4]. In 2005, Wolfe ND [5] and Calattini S [6], reported the discovery of the third and fourth HTLV types (HTLV type 3 and 4) in asymptomatic Cameroonese hunters. Some studies had revealed Western blot profiles compatible with HTLV-1 and HTLV-2 in those individuals suggesting an appreciable cross-reaction between these viruses [5], [7].

HTLV-1 has a remarkable genetic stability when compared with other retroviruses such as HIV (Human Immunodeficiency Virus). However, based on the nucleotide diversity of its LTR region, HTLV-1 can be grouped in 6 major genetic subtypes (a–f), most of them linked to some geographic regions [5], [8]–[12] All subtypes are present in Africa with different prevalences, except for HTLV-1c that has, so far, only been identified in Melanesia [13]. The genetic lineages of HTLV-1a subtype, also known as Cosmopolitan group, are found all over the world. This group can be further divided in distinct sub-groups, which are characteristics of geographical localization of HTLV-1 infections. The HTLV-1a Cosmopolitan subgroups identified as driving the HTLV infection in Africa were HTLV-1aD or North African subgroup, which is prevalent in Senegal, Guinea Bissau, Morocco, and the transcontinental subgroup in South Africa [14]. HTLV-1 is the etiological agent of Adult T-Cell Leukemia/Lymphoma (ATL) and Tropical Spastic Paraparesis/HTLV-1-associated Myelopathy (TSP/HAM). Efforts to disrupt its transmission have been taken in some countries, including the screening of blood donors for the presence of HTLV antibodies.

HTLV screenings performed in many African countries have been limited to sero-epidemiological surveys studies using ELISA and Western Blot criteria for HTLV typing [15]–[16]. Recently, the epidemiology of this virus was addressed in donors attending the blood bank of the Maputo city showing rates from 0.9 to 1.2% [17]–[18]. Additionally, a study conducted in 1999 showed that four TSP/HTLV patients from Mozambique were infected with Cosmopolitan HTLV-1 subtype A [19]. It has recently been shown that the prevalence of HTLV among HIV-infected patients in Mozambique is 4.5%, much higher than in blood donors [20]. Mozambique is one of the countries in sub-Saharan Africa with the highest HIV prevalence rates in adult population. The HIV seroprevalence among pregnant women in Mozambique rise in the proportion of infected women of 14.0% in 2002, 17.8% in 2003, 16.5% in 2004, and 20.2% in 2005 [21]. In another serosurvey performed in 2007, a prevalence of HIV infection around 18% was found in women attending antenatal services [22]. HIV-1 subtype C is the major variant of the HIV/AIDS epidemic in the country [23]. However, the status of HIV and HTLV co-infections in Mozambique is still poorly documented, as well as, the subtypes/subgroups of these viruses.

The aim of this study was to investigate the molecular identities of HTLV in Mozambique and therefore to gain insights to HTLV infection in the country, focusing on a set of HTLV seropositive blood donors and HTLV/HIV co-infected individuals from Maputo city.

## Methods

### Ethics Statement

Both Co-infection Protocol (148/CNBS/05) as well the Blood Bank Study (78/CNBS/06) were reviewed by National Center in Bioethics in Health (CNBS) located in MOH, Maputo, Mozambique. All subjects provided written informed consent and were included in the study.

### Samples

HTLV-1 positive samples were originated from a cross-sectional study conducted among 2019 repeat blood donors at the Maputo Central Hospital blood bank in Maputo city between August and December 2006, where the prevalence of the infection is 0.9% [18]. HTLV/HIV positive samples were originated from a cross-sectional survey conducted among HIV-positive adult individuals naive to Highly Active Antiretroviral Therapy (HAART) attending an HIV Outpatient Clinic in Maputo, between March and June of 2006, with prevalence of HTLV/HIV co-infection of 4.5%. Demographic information is on Gudo et al. [18]. All twenty-five specimens, 14 from blood donors and 11 from HTLV/HIV co-infected individuals, were positive by HTLV enzyme immunoassays [Murex HTLV I+II, (Abbott/Murex, Wiesbaden, Germany) and Vironostika HTLVI/II (bioMérieux bv, Boxtel, Netherlands], and confirmed by HTLV BLOT 2.4 (Genelabs Diagnostics, Singapura). The HIV positive individuals were screened by two rapid tests: Determine HIV 1/2 test (Abbott Laboratories, Tokyo, Japan) and Unigold HIV test (Trinity Biotech, Ireland).

### Genetic analysis

High molecular weight DNA was extracted from whole-blood samples of all 25 subjects using QIAamp DNA Blood Mini kit (Qiagen). The PCR for HTLV typing was carried out as described [24]. The HTLV-1 subtyping was performed with amplification of 672 bp fragment from the LTR region under conditions published previously [25] using *Pfu* DNA polymerase (Stratagene). Human mitocondrial (mt) DNA hypervariable segment I (HVS-I) (302 bp) was accessed by PCR using conditions formerly described [26]. The amplicons were separated on a 2% agarose gel and visualized under UV light after ethidium bromide staining. PCR products corresponding to HTLV-1 LTR and mtDNA HVS-I regions were purified using QIAamp PCR purification kit (Qiagen) according to the manufacturer's instructions. The amplicons were directly sequenced on both strands using the BigDye Terminator v3.1 Cycle Sequencing Kit, with a 3730 Automated DNA Sequencer (Applied Biosystems, USA) and the bioinformatics pipeline Chromapipe [27].

LTR sequences were editing and alignment using BioEdit v5.0.9. (Department of Microbiology, North Carolina State University, USA) and Clustal W programs, respectively. Neighbor-joining (NJ) tree was build by PAUP* software version 4.0b10 [28]. The HKY model with gamma distribution was selected using the Modeltest 3.7 software [29]. The NJ tree was evaluated by bootstrap analysis of 1000 replicates. The mean genetic distance among the Mozambican HTLV-1 clusters were determined using nucleotide p-distance model and standard error estimated by a bootstrap procedures (1000 replicates) on Mega4 software [30]. Mozambican HTLV-1 sequences are available at GeneBank (accession number GU194504-GU194528).

The mtDNA sequences were classified in haplogroups in agreement with the HSV-I nucleotide polymorphisms described [26], [31]–[32]. The HVS-I nucleotide sequences are available at GeneBank (accession number FJ888491-FJ888504; HM775388- HM775395).

## Results

**Figure 1:**
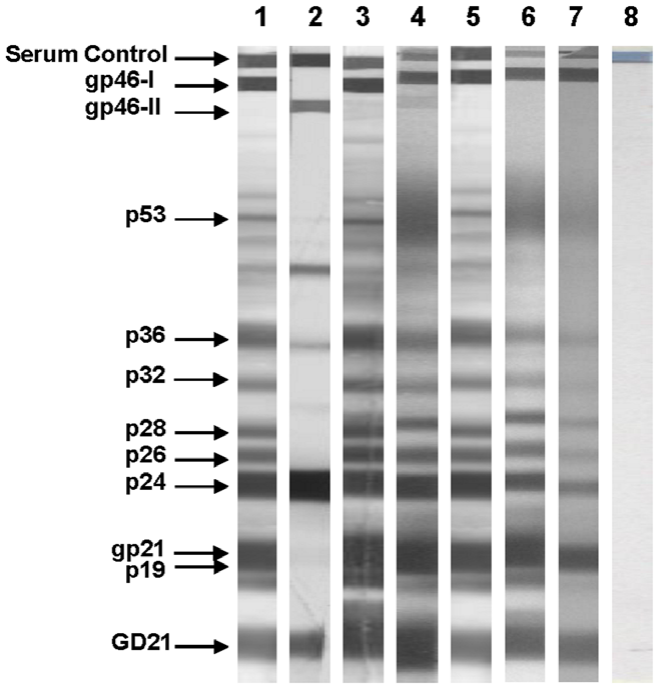
WB patterns of Mozambican individuals infected by HTLV-1. Lane 1: HTLV-1 positive control; lane: 2 HTLV-2 positive control; lane 3–7: plasma samples; lane 8: HTLV-1/2 negative control.

The results of screening for anti-HTLV-1+2 antibodies and confirmed by a Western blot assay (HTLV BLOT 2.4, Genelabs Diagnostics, Switzerland) are in Figure 1. Most of blood donors with reactivity to antigens encoded by the *gag* and *env* genes were considered as infected by HTLV, according to the instructions provided by the manufacturer. Two samples were negative to p53 band and one to P19. All HTLV positive samples in our study population were reactive to rgp46-HTLV-I and typed as solely infected with HTLV-1 by Western blot with no reaction to recombinant rgp46-HTLV-II.

**Figure 2:**
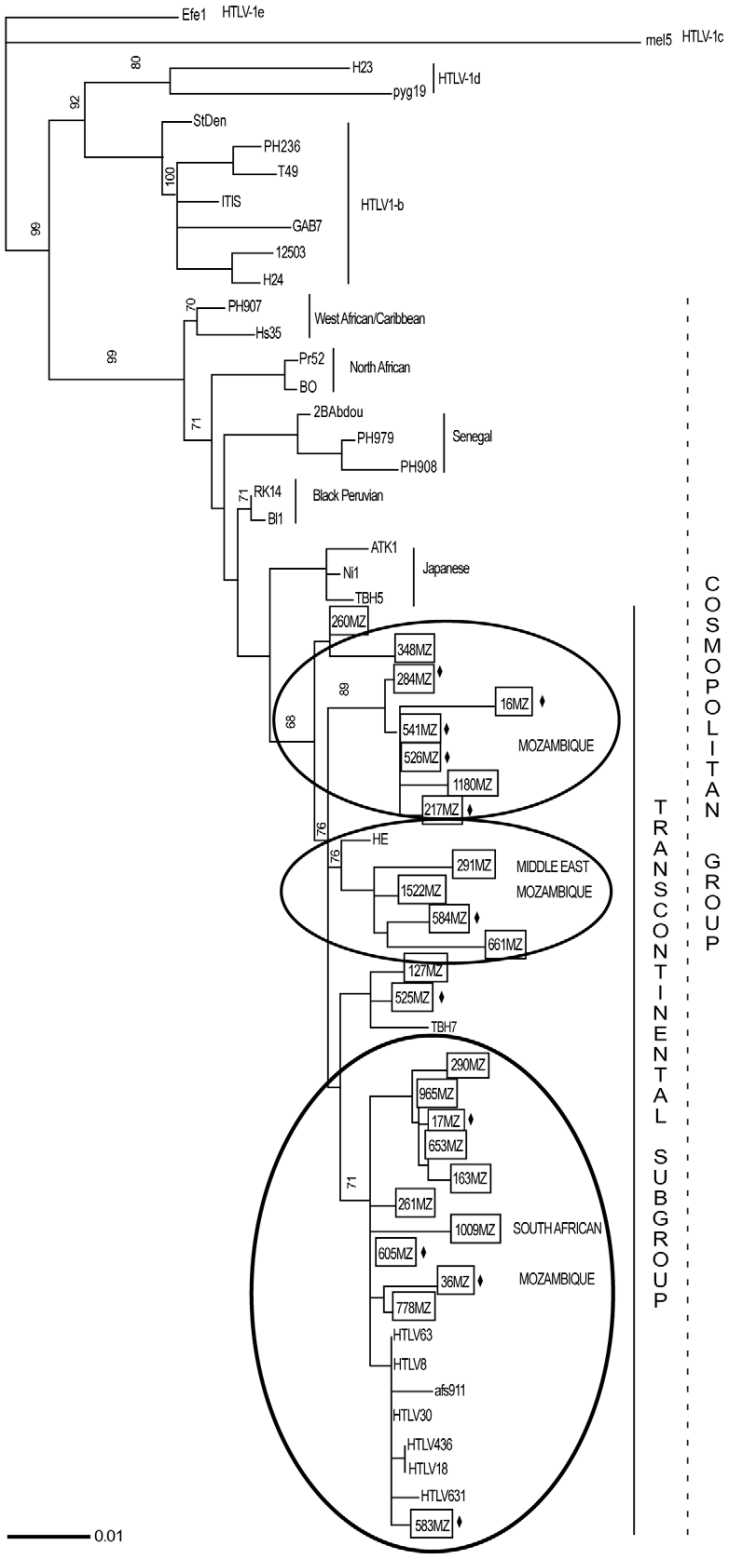
Genetic Analysis of Mozambican HTLV-1 LTR strains. HTLV-1 LTR (630 bp) genetic tree of 25 Mozambican isolates with HTLV-1 references and worldwide sequences were constructed using Neighbor Joining method based on HKY model and γ-distribution. Bootstrap values were calculated by the analysis of 1000 replicates. Only bootstraps values >50 are shown. HTLV strains from this study are in boxes and HIV-1 co-infected samples have a black losange. HTLV-1a sub-clusters are in a circle.

The genetic analysis of 25 Mozambican LTR sequences, using 32 reference sequences representing all HTLV-1 subtypes and sub-groups, as well as African HTLV-1 sequences, clearly demonstrated that all isolates belong to the Transcontinental subgroup of the Cosmopolitan subtype (Figure 2). These new sequences from Mozambique showed an inter-strain genetic distance ranging from 0.0 to 3.4% with an overall mean distance of 1.6% and standard error (SE) estimated of 0.3. This variability is reflected in the grouping of Mozambican LTR sequences in three major clusters (Figure 2). Thirteen from 25 Mozambican (MZ) sequences are close together with sequences from South Africa (SA) forming a consistent branch, supported by a bootstrap value of 71%. This cluster, named MZ/SA, has an inter-strain genetic distance of 0.0–2.1% (01.1%; SE = 0.2) and is characterized by the polymorphism C386T (ATK reference sequence). Four sequences with inter-strain genetic distance ranging from 1.0–2.1%, mean diversity of 1.4% (SE = 0.3) are related with the sequence HE (GenBank S76263) from Israel, which belongs to Middle East cluster as showed by Ohkura and others, 1999. Another genetic analysis including more HTLV-1 sequences from this geographical region, based on 490 pb, showed that those 4 Mozambican sequences forming a monophyletic group with sequences from Middle East (ME): KUW3, SAS, IRN4, Abl and India: AP15, TNA (data not shown). The cluster named MZ/ME, is supported by a bootstrap value of 76% and sequences are characterized by a 6 nt deletion between 182–186 nt positions and present the substitutions A240G and A575C. Of note, 6 Mozambican distinct sequences (16 MZ, 217 MZ, 526 MZ, 1180 MZ, 284 MZ, 541 MZ) with mean genetic distance of 0.8% (0.0–01.8%; SE = 0.2) determined the MZ cluster with a high bootstrap support of 89% and characterized by three nucleotide changes: C365T, T573C, and C574T. Some of these HTLV infected individuals (11/25 = 44.0%) were HIV-1 co-infected (Figure 1). In fact, the HIV-1 subtype found in all HTLV co-infected individuals, the HIV-1 subtype C, is the major HIV subtype circulating in Maputo, as shown by Abreu et al., 2008 [23]. A slight correlation of HIV/HTLV co-infection with cluster MZ could be observed (5/6 = 83.3%). MZ/ME and MZ/SA clusters have HIV/HTLV co-infection frequencies of 25.0% and 45.4%, respectively (Figure 2).

In order to check the representativeness of this sampling in relation to Mozambican population, mtDNA haplogroup analysis was conducted. This analysis, based on HSV-I sequence motifs, revealed that all those Mozambican individuals belong to African macrohaplogroup L, including the sub-haplogroups L1a, L1c, L1d, L2a, L3d and L3e. The African haplotypes were detected at different frequencies of L1a (28%), L1c (8%), L1d (12%), L2a (36%), L3d (12%), L3e (4%) and indistinctly distributed within the 3 HTLV-1 LTR clusters described above.

## Discussion

The two pandemic human retroviruses, HIV and HTLV, were originated as zoonoses in Africa at very distinct times. HTLV is an ancient virus while HIV emerged in the early 20th century [33]–[34]. The present study characterized the HTLV-1 strains in blood donors and HTLV/HIV co-infected individuals from Maputo city, Mozambique, as well as, determined the genetic background of those individuals.

The HSV-I mtDNA haplogroup analysis from HTLV-1 infected individuals revealed that all the Mozambican sequences belong to African sub-Saharan haplogroups. It was not possible to establish a correlation between HTLV-1 lineages and a specific ethnics group, since diverse mtDNA haplotypes were observed in the three HTLV-1LTR clusters. All African sub-haplogroups detected in this study (L1a, L1c, L1d, L2a, L3d and L3e) were reported as the major frequencies in Mozambique [21]; [25]. In fact, sub-haplogroups L2a and L1a were detected at the highest frequencies (36% and 28%, respectively), as reported in previous studies of Mozambican population. An mtDNA analysis of 109 unrelated individuals, corresponding to 30 ethnic groups, showed the L2a and L1a sub-haplogroups with the highest frequencies of 47% and 16%, respectively [21]. A larger analysis, using 307 samples from 16 different population groups from Mozambique and boundary areas, also revealed the same haplogroups distribution, with L1a and L2a (28% and 27%, respectively) as the major sub-haplogroups, differently from other geographical regions in Africa [35]. Haplogrup composition and their frequencies are characteristic of main African regions. In South Africa, there is a predominance of mtDNA haplogroup L1d/k, but L2a and L1a are in low frequencies. Central and West Africa, also present high frequencies of haplotypes [L1c and L2* (L2 other than L2a1), respectively], which in Mozambique is less representative [35]. Therefore, we can conclude that the Mozambican HTLV-1 cohort considered in our study is representative of the country population.

The sub-Saharan Africa is considered to be endemic for HTLV-1 infection, with overall seroprevalence rates in Western and Central African countries being similar to the ones observed in Eastern and Southern African countries [17], [18], [36]–[38]. However, there is lack of data concerning the HTLV molecular epidemiology in Eastern African countries, contrasting with studies in Western countries and South Africa [14], [39]. In spite of HAM/TSP cases have been reported in Africa [3], including Mozambique [19], there is no information on the clinical impact of HTLV-1 in African population. The risk of HTLV-I-associated diseases among carriers differs substantially across geographic areas and according to other population characteristics. ATL is reported to be prevalent among individuals in Japan, while Brazilian patients tend to present the HAM/TSP disease, and in Jamaica these two HTLV-1 related illness are quite frequent, suggesting that particular genetic backgrounds may play a role in the disease development [40].

Most of the genetic variability of HTLV-1 is represented in Africa, where the virus was originated [41]. Mozambique is located on the southeast region of the continent, and the presence of HTLV-1 has also been reported in countries that share borders with Mozambique [40]. The characterization of HTLV-1 Transcontinental subgroup as the prevalent subgroup driving the HTLV epidemics in Mozambique, and South Africa [38] shows that there are, at least, two major endemic lineages of Cosmopolitan HTLV-1 in Africa. One, dominating the Northwestern part of the continent, driven by HTLV-1aD trans-Saharan lineage, as proposed by Zehender [14] and a second lineage in Southern countries, determined by Transcontinental HTLV-1 subgroup.

The genetic relationship of HTLV-1 Transcontinental subgroup strains from South Africa and Mozambique suggests a common origin of HTLV-1 in both countries, probably due to their common peopling and migration patterns and their intense commercial/migratory linkage and vicinity. It was interesting to find a cluster joining Mozambique, Middle East and India strains that can also be explained by a noticeable Indian population in Maputo city due to the Indians people migration started in the 19^th^ century as well as, consequence of the migratory movements between India and Austral Africa (1860) [42]. However, the characterization of a cluster defined only by Mozambican strains suggests the existence of HTLV-1 focus in the country. In fact, the reproduction of Mozambican mtDNA haplogroup pattern on the HTLV-1 cohort analyzed in this study, may explain the particularities of HTLV-1 infection in the country. Mozambican mtDNA haplogroup pattern have components from North and South, with a high frequency of moderns haplogroups (L2), haplogroups implicated in Bantu expansion (L1a and L3), but also the ancient haplogroup L1d, characteristic of the extinct Khoisan-speaking ethnic group [31], [35], is present. Due to the diversity of mtDNA haplogroup composite, we could speculate that both ancient and recent introductions of HTLV-1 infection are responsible for the current picture, modulates by breastfeeding and sexual/injecting drug transmission ways, respectively. Of concern, the significant rate of HTLV-1a/HIV-1subtype C co-infection, particularly in the MZ cluster, demonstrates the dynamic of the two viruses. In fact, the HIV-1/HTLV-1 co-infection posses a great challenge in AIDS follow up tests such as CD4 enumeration related to the prognosis [18], and the need for implementation of public health control measures, as well clinical protocols focusing on both HIV-1 and HTLV-1 in Mozambique.
